# An ultrastructural study of the surface and attachment structures of *Paradiplozoon homoion* (Bychowsky & Nagibina, 1959) (Monogenea: Diplozoidae)

**DOI:** 10.1186/s13071-017-2203-8

**Published:** 2017-05-25

**Authors:** Veronika Konstanzová, Božena Koubková, Martin Kašný, Jana Ilgová, Ewa Dzika, Milan Gelnar

**Affiliations:** 1 Department of Botany and Zoology, Faculty of Science, Masaryk University, Kotlářská 2, 611 37 Brno, Czech Republic; 2 Department of Parasitology, Faculty of Science, Charles University, Viničná 7, 128 44 Prague, Czech Republic; 3 Department of Medical Biology, Faculty of Medical Sciences, University of Warmia and Mazury in Olsztyn, Żolnierska 14c, 10-561 Olsztyn, Poland

**Keywords:** *Paradiplozoon homoion*, Ultrastructure, Neodermis, Tegument, Attachment clamps

## Abstract

**Background:**

Species of *Diplozoon* Palombi, 1949 (Monogenea: Diplozoidae) are blood-feeding ectoparasites mainly parasitising the gills of cyprinid fishes. Although these parasites have been the subject of numerous taxonomic, phylogenetic and ecological studies, the ultrastructure of the surface and haptor attachment structures remains almost unknown. In this study, we used transmission electron microscopy to examine the ultrastructure of attachment clamps and neodermal surface of *Paradiplozoon homoion* (Bychowsky & Nagibina, 1959), family Diplozoidae Palombi, 1949, thereby broadening our knowledge of platyhelminth biology.

**Results:**

The hindbody surface of *P. homoion* is distinctly ridged, each ridge being supported by several muscle fibers and equipped with scales on the surface plasma membrane. Such structures have not been recorded previously in species of the family Diplozoidae. Comparisons of the surface structure of different body parts revealed slight differences in the thickness and number of organelles. Each of the clamps has a flattened bowl-like structure composed of sclerites, movable skeletal-like structures that are anchored by robust, radially oriented muscle bundles. The base of the posterior median plate sclerites is equipped with glandular cells possessing secretory vesicles.

**Conclusion:**

This study brings detailed ultrastructural data for the surface and haptoral attachment clamps of *P. homoion* and provides new insights into the ultrastructure of Diplozoidae. Glandular cells at the base of the attachment clamps responsible for sclerite development in diplozoid species were observed for the first time. Our findings support the hypothesis that the structure of particular neodermal compartments is similar within the Platyhelminthes. On the other hand, the diplozoid glandular system and the mechanism of sclerite development clearly merits further attention.

## Background

Species of *Diplozoon* Nordmann, 1832 are blood-feeding ectoparasites mainly parasitising the gills of cyprinid fishes, where they can cause mechanical damage to the gill filaments, initiating the development of secondary infections (bacterial, mycotic) and anemia [1, 2]. During the diplozoon life-cycle, two larvae (diporpa) pair and subsequently fuse permanently, producing the typical X-shaped diplozoon body arrangement, a characteristic unique to the Diplozoidae Palombi, 1949 [3]. Diplozoon spp. have developed a number of adaptations for successful attachment to gills, including central hooks and clamps located on the posterior adhesive organ, the haptor. The neodermis (tegument) in these species is also associated with a number of surface structures that play an important role in the ectoparasitic life-style. The neodermis has been investigated at the ultrastructural level in other monogenean species, including *Gyrodactylus* sp. [4, 5], *Entobdella soleae*, *Acanthocotyle elegans* [6], *Diclidophora merlangi* [7], *Rajonchocotyle emarginata*, *Plectanocotyle gurnardi* [8], *Diplectanum aequans* [9], *Allodiscocotyla diacanthi* [10], and *Metamicrocotyla macracantha* [11]. Studies on species within the Diplozoidae, however, are missing. In general, the external surface of monogeneans is a syncytium formed by neodermal (tegumental) cells. The distal cytoplasm is connected *via* cytoplasmic junctions with the proximal cytoplasm of cell bodies located in the subsurface parenchyma [7, 12–14].

An important part of the diplozoid monogenean body is the complex of posterior attachment structures on each opisthaptor, comprising a pair of central hooks and four pairs of clamps [15, 16]. These allow the parasite to attach to the gills using an extrinsic muscle/tendon system associated with a median J-shaped sclerite [17]. The clamps comprise a posterior and anterior jaw, joined with a median plate by anterior and posterior joining sclerites [18, 19]. These clamps are morphologically distinct and can be used for species determination [19–22]. While the ultrastructure of heteronchoinean clamps has been investigated previously [23–28], ultrastructural data for diplozoids are presently missing. In general, the clamp wall surrounding the inner sclerites is formed of a specialized muscle complex with radially oriented muscle fibers [22]. Chemical analysis has shown that heteronchoinean sclerites, including those in *Diplozoon paradoxum*, consist of scleroproteins [29–32].

The main aim of this study was to undertake the first detailed ultramicroscopic analysis of the neodermal surface and attachment structures of *Paradiplozoon homoion* Bychowsky & Nagibina, 1959, a generalist parasite infecting a range of cyprinid fish species, including roach *Rutillus rutilus* (L.), bleak *Alburnus alburnus* (L.) and gudgeon *Gobio gobio* Fleming [33, 34]. The species is an oviparous monogenean parasite with a relatively high prevalence in European populations of wild and farmed fish. Our paper forms part of a complex study focusing on diplozoid morphology and ultrastructure and phylogenetic and functional indicators for their specialized life strategy.

## Methods

### Sample origin and collection

Adult *P. homoion* were collected from the gills of bleak caught in the littoral zone of the Mušov lowland reservoir (Czech Republic; 48°53′12″N, 16°34′37″E) in 2013. The fish were transported live in oxygenated water to the parasitological laboratory at the Faculty of Science, Masaryk University, where they underwent a standard parasitological autopsy [35] modified for the detection of diplozoid species, i.e. only the gills were examined. After euthanasia, the gills were extracted and checked for the presence of all diplozoid ontogenetic stages under an Olympus SZX 7 zoom stereo microscope. Live worms were determined based on their morphology examined under an Olympus BX50 light microscope equipped with Nomarski differential interference contrast and an Olympus Stream Motion digital image analysis system v. 1.9.2. The parasites were then processed and fixed for further light and transmission electron microscopy analysis.

### Transmission electron microscopy

Parasites intended for transmission electron microscopy (TEM) were washed three times in freshwater to remove any remaining mucus. Live specimens were fixed directly in 2% osmium tetroxide for 1 h and dehydrated through an ascending acetone series. The dehydrated samples were immediately embedded in Spurr resin [36]. In order to obtain a better orientation in basic diplozoon morphology, ten longitudinal and ten transversal semi-thin sections (0.5 μm) of whole worm bodies were cut (from the anterior extremity of body through the pharynx to the posterior extremity) using a Leica EM UC6i ultramicrotome. Sections were stained with toluidine blue for 30 s at 80 °C to contrast the acidic components (sulfates, carboxylates and phosphate radicals) of the different tissues [37]. Selected ultrathin sections were contrasted with uranyl acetate lead by citrate and examined using a JEOL JEM-1010 TEM operating at 60 kV. Images were taken for further analysis using Megaview II software (ResAlta Research Technologies).

## Results

### General morphological characterization

**Fig. 1:**
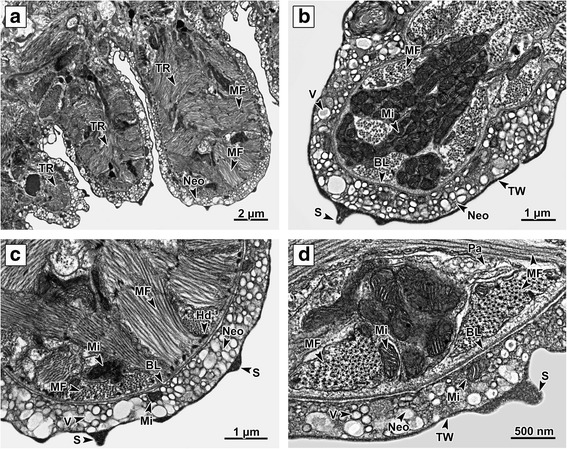
Body surface ridges. **a** Longitudinal section through the body surface transverse ridges in the cross-body area. The transverse ridges are supported by numerous multi-directional muscle fibers. **b** Detailed view through a ridge. The surface of each ridge is covered by a syncytial layer containing numerous vesicles. Scales are located on the external neodermis plasma membrane. Beneath the membrane, the cytoplasm is more electron-dense and fibrous, forming a terminal web. Numerous muscle fibers and mitochondria are located under the basal lamina. **c** Muscle fibers, attached to the basal lamina by the hemidesmosomes. Note the muscle fibers in different directions. **d** Detailed view of the apical part of the ridge. Note (i) the space between the muscle fibers is filled with parenchyma; (ii) the presence of numerous mitochondria with well-developed cristae; (iii) the outer syncytial layer contains different vesicle types and mitochondria; and (iv) the scale connected to the distal plasma membrane. *Abbreviations*: BL, basal lamina; Hd, hemidesmosomes; Mi, mitochondria; MF, muscle fibers; Neo, neodermis; Pa, parenchyma; S, scales; TR, transverse ridges; TW, terminal web; V, neodermal vesicles

The body of *P. homoion* is covered with a neodermal syncytium consisting of multinucleated tissue with no distinct cell boundaries. The hindbody surface is distinctly ridged, with obvious transverse 15 × 8 μm annular ridges located especially in the space between the cross-body and the clamps (Fig. 1a-d). Each of the ridges is supported by muscle fibers lying under the basal lamina and is equipped with scales on the surface plasma membrane (Fig. 1c). The apical part of the ridge inner space, proximal to the basal lamina, is filled with numerous clusters of oval mitochondria with well-developed cristae (Fig. 1b-d). The haptoral clamps, comprising sclerotized elements (sclerites) bounded by muscle tissue, are located on the basal part of the diplozoon hindbody (Fig. 5a-f).

### Neodermis

**Fig. 2:**
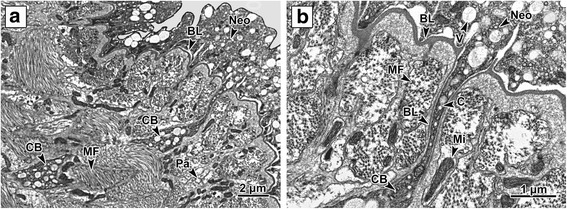
Cytoplasmic bridge. **a** The outer syncytial layer located externally to the basal lamina covers whole diplozoon body. The inner nucleated bodies and outer syncytial layer are interconnected by the cytoplasmic bridge. **b** Cytoplasmic bridge in detail. The bridge is delimited by the basal lamina and filled with cytoplasm. Numerous muscle fibers and mitochondria are located proximally to the basal lamina. The cytoplasm of external syncytial layer is filled mainly by numerous different granules. *Abbreviations:* BL, basal lamina; C, cytoplasm; CB, cytoplasmic bridge; MF, muscle fibers; Neo, neodermis; Pa, parenchyma; V, neodermal vesicles

**Fig. 3:**
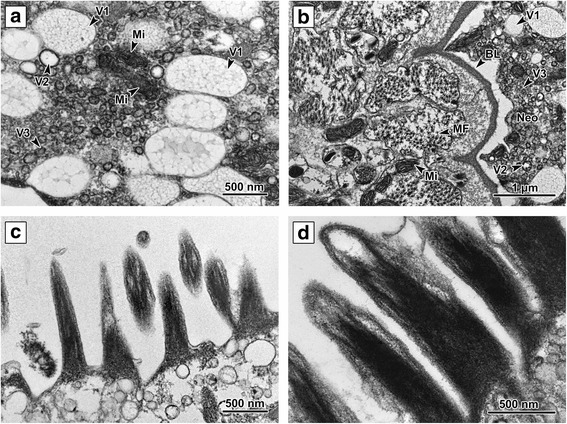
Ultrastructure of the outer layer. **a** The cytoplasm of the outer syncytial layer is filled with different vesicle types. Small groups of mitochondria with few cristae are scattered between the vesicles in the outer syncytial layer space. **b** The outer syncytial layer, with vesicles, on the distal side of the basal lamina. Several layers of circular and longitudinal muscle fibers are located proximally to the basal lamina. Basal lamina protuberances directing proximally to the subsurface area; the protuberances denoting the presence of cytoplasmic bridges connecting the nucleated cell bodies and the external syncytial layer. **c** External surface of mouth cavity neodermal syncytial layer covered with microvilli. **d** Detail of the microvilli; these are covered by plasma membrane and have a dense bundle which serves as a structural core. *Abbreviations*: BL, basal lamina; Mi, mitochondria; MF, muscle fibers; Neo, neodermis; V1, V2, V3, vesicle types

The *P. homoion* neodermis (tegument) comprises two main parts, an outer syncytial layer located distally to the basal lamina and an inner layer containing the nucleated bodies of the neodermal cells. The inner bodies are located proximally to the basal lamina below the body wall musculature (Fig. 2a, b). The inner bodies are irregularly shaped and contain large nuclei with a nucleolus, the cytoplasm containing vesicles and scattered mitochondria. The nucleated bodies are connected to the outer layer *via* cytoplasmic bridges perpendicular to the body surface. The inner bodies and their cytoplasmic bridges were rather scarce and not straight; hence, it was not possible to obtain a complete longitudinal section (Fig. 2a, b). The outer syncytial layer is delimited by the surface and basal plasma membranes and is mainly filled with different types of inclusions. The surface plasma membrane of the neodermal syncytium is covered with external scales, mainly on the hindbody ridges (Fig. 1b). Immediately beneath this membrane, the cytoplasm is more electron-dense and fibrous, forming a region that can be interpreted as a terminal web. The syncytial cytoplasm contains three types of vesicle, a large spherical vesicle (800 nm in diameter) with fibrous, moderately electron-dense to translucent contents (V1), a small spherical vesicle (250 nm in diameter) with homogeneous electron-translucent contents (V2), and a very small spherical vesicle (100 nm in diameter) with electron-opaque homogeneous contents (V3) (Fig. 3a, b). All vesicle types were located in both the cytoplasmic bridge space and the external layer (Fig. 2a, b). The clusters found in the outer syncytial layer space comprise rounded mitochondria with few cristae surrounded by the outer mitochondrial membrane (Fig. 3a). The basal lamina, consisting of two layers (lamina densa and lamina lucida), lies immediately beneath the basal plasma membrane and has a uniform thickness and numerous deep folds (Figs. 2a, 3b).

The musculature lying proximally to the basal lamina is well developed and formed by several layers of longitudinal and circular muscle fibers, with the circular muscle fibers placed proximally to the basal lamina (Fig. 3b). All muscle fibers are attached to the internal side of the basal lamina by hemidesmosomes (Figs. 1c; 5d, f). The space between the muscle fibers is filled with interstitial material and the fibers themselves are bound by sarcolemma.

**Fig. 4:**
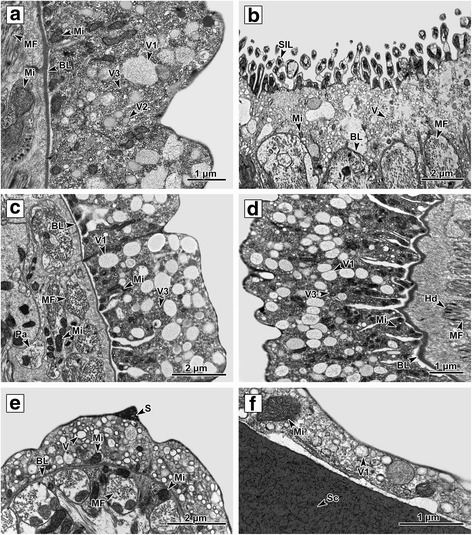
Variation in the outer layer from different body parts. **a** Anterior part of the forebody. **b** Apical part of the mouth cavity; note the layer with microvilli directing toward the mouth cavity lumen on the external surface. **c** Middle part of the forebody. **d** Cross-body area. The syncytial cytoplasm is filled with numerous mitochondria and a considerable number of V1 vesicles. **e** Middle part of the hindbody ridged with a few scales located on the distal surface. **f** Clamp outer syncytial layer. *Abbreviations*: BL, basal lamina; Hd, hemidesmosomes; Mi, mitochondria; MF, muscle fibers; Neo, neodermis; Pa, parenchyma; V1, V2, V3, vesicle types; S, scale; Sc, sclerite; SIL, microvilli; V, neodermal vesicles

The outer syncytial layer covering the anterior forebody is usually of medium width (diameter 3–4 μm), while that covering the apical part of the mouth cavity is 3–7 μm in diameter and equipped with microvilli on its external surface (Figs. 3c, d and 4a, b). The microvilli are covered by plasma membrane and have a dense bundle which serves as its structural core. The surface on the middle forebody is similar to that at the anterior forebody, being of medium-width (*c*.4 μm diameter) and having a smooth, slightly undulating surface (Fig. 4c). The cross-body area is covered with a thick (*c*.6 μm wide) outer layer with an undulating surface (Fig. 4d). This is the thickest part of the *P. homoion* surface external layer. The middle hindbody external layer is ridged and is covered with a thin, smooth outer syncytial layer with maximum width of 1–2 μm with a few scales on the external side (Fig. 4e). The outer syncytial layer covering the clamps is regular, smooth on its surface and very thin, with a maximum width of 0.75 μm. This is the thinnest part of the external surface layer (Fig. 4f). Large mitochondria (500 μm in diameter) were detected in the cytoplasm of the clamp outer layer. The outer syncytial layer on the middle forebody and cross-body region contains a higher proportion of V1 vesicles (Fig. 4c, d). This is in contrast to the other body parts, where the V2 and V3 vesicles tend to predominate (Fig. 4a, b, e, f).

### Clamps

**Fig. 5:**
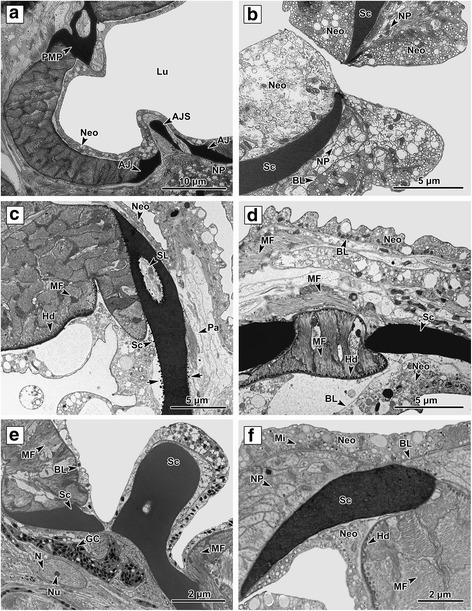
Ultrastructure of clamp I. **a** The central part of two anterior jaw sclerites connected by an anterior joining sclerite and the posterior part of a median plate sclerite. The nerve fibres are located at the base of the anterior joining sclerite. **b** Terminal part of both jaws forming the clamp, i.e. the part coming in direct contact with host tissue (gills). **c** A cross-section of the clamp musculature, with hemidesmosomes connected to the lateral sclerite; note also the electron-dense material (*arrows*) adherent sclerite surface. **d** A detail of the sclerite surrounded by radial muscle fibers with clearly visible hemidesmosomes. **e** A detail of the posterior median plate sclerite; note the glandular cell with secretory vesicles and a nucleus with nucleolus in the cytoplasm at the basal area of the sclerite. Secretory vesicles are spread throughout the syncytial layer cytoplasm. **f** A detail of the anterior jaw sclerite, showing the large nerve plexus proximally to the external syncytial layer and the well-developed musculature, enabling clamp movement. *Abbreviations*: AJ, anterior jaw sclerites; AJS, anterior joining sclerite; BL, basal lamina; GC, glandular cell; Hd, hemidesmosomes; Lu, luminal part of the clamp; MF, muscle fibers; N, nucleus; Neo, Neodermis; Nu, nucleolus; NP, nerve fiber; PMP, posterior part of median plate; Sc, sclerite; SL, sclerite lumen

**Fig. 6:**
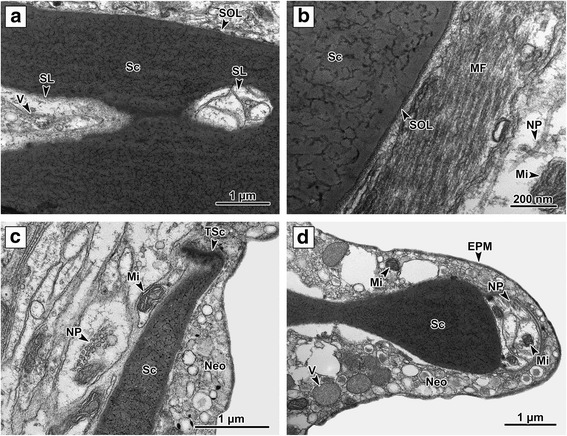
Ultrastructure of clamp II. **a** A clamp sclerite limited distally by the outer layer, with a sclerite lumen inside. **b** A section through the muscle fibers enabling sclerite movement. The muscle fibers are attached to the sclerite on its outer layer. **c** Terminal part of the sclerite with its hooked tip. **d** Detail of the terminal part of the anterior joining sclerite. A large nerve fiber is located on the terminal part of the sclerite. *Abbreviations*: EPM, external plasma membrane; Mi, mitochondria; MF, muscle fibers; Neo, neodermis; NP, nerve fiber; Sc, sclerite; SL, sclerite lumen; SOL, outer layer; TSc, terminal part of the sclerite; V, vesicles

Each of the clamps has a flattened bowl-like shape, the structure comprising sclerites and movable skeletal-like structures anchored by robust, radially oriented muscle bundles (Fig. 5a-f). The clamps are covered by a thin neodermal syncytial layer over the whole surface. This layer is thinner than that covering the rest of the body (Fig. 6c, d).

Each sclerite is closely bound by the basal lamina of the surrounding muscle complex (Fig. 6b). Between the body of the sclerites and the surrounding basal lamina there is a space filled with closely packed fibrous content known as the outer layer (Fig. 6b). The sclerite body is composed of a moderately electron-dense material containing fibrils, which are irregularly organized and of different lengths; many of them are branched, forming randomly orientated three-dimensional fibrous structures (Fig. 6a, b). Large cavities with closely packed fibrils, mitochondria and numerous small electron-dense granules inside the lumen are clearly visible in longitudinal sections through the sclerites (Fig. 6a). Nerve fibers were located relatively close to the terminal and basal parts of sclerites (Figs. 5a, b, d and 6c, d). Very small (0.1 μm in diameter) electron-dense, membrane-bound vesicles were detected adhering to the external surface of the sclerites (Fig. 5c). Some vesicles were also located in the parenchyma space and inside sclerites on the internal surface of the cavities.

The base of the posterior median plate sclerites is equipped with glandular cells possessing secretory vesicles (Fig. 5e). The same secretory vesicles are located in the surface external syncytial layer covering the medial plate sclerite.

## Discussion

### General morphological characterization

Comparison with previous studies concerning platyhelminth surface structures [38–40] confirms that *P. homoion* share analogous morphological characteristics with other Platyhelminthes. While there have been numerous previous studies focused on the morphology and ultrastructure of monogenean surface and attachment structures using TEM [10, 11, 23, 24, 26, 27, 41], none have included species from the family Diplozoidae. Highly developed transverse tegumentary annular ridges were identified on the hindbody of *Eudiplozoon nipponicum* using scanning electron microscopy [42]. The same ridges were later recognized on histological sections and termed “tegumental folds” [43]. Our findings on *P. homoion* suggest that such ridges are not solely of outer neodermal (tegumental) syncytium origin, the ridges comprising well-developed musculature, the surface of which is covered with an outer syncytial layer. The outer surface layer of the hindbody ridges is equipped with several electron-dense scales on the external plasmatic membrane. Epidermal scales were also recorded on the posterior surface of the monogenean *D. aequans* [9]. These scales, which were composed of moderately electron-dense material, were located within the tegumental external syncytial layer cytoplasm, beneath the outer membrane. Relatively large and apparently hard scale-like neodermal structures have been reported as ‘spines’ or ‘scales’ in the monogenean *Pseudorhabdosynochus* spp. (Diplectanidea) [44], but these were not examined at an ultrastructural level. While the function of the scales on *P. homoion* is still not fully understood, their presence could suggest a protective role.

### Neodermis

The primary surface structure of *P. homoion* is very similar to that of other monogenean species, e.g. *D. merlangi* [7]; *R. emarginata*, *P. gurnardi* [8]; *Pricea multae* [25]; *Atriaster* sp. [45]; *Tetraonchoides* sp. [46]; *M. macracantha* [11]; *Dictyocotyle coeliaca* [47]. In each of these, the outer syncytial layer is connected to the nucleated cell body *via* cytoplasmic bridges.

Different granular and vesicular bodies have been observed in the syncytial layer cytoplasm in all previously studied monogeneans [12, 26, 45, 48, 49]. These bodies are produced by Golgi stacks in the inner nucleated regions and exported to the outer syncytial layer. This corresponds well with our TEM results for *P. homoion*, whereby the vesicles were present in both inner bodies and the outer layer. Unlike *Gotocotyla bivaginalis* [26], for which four vesicle types were documented in the outer cytoplasm layer, we observed only three types in *P. homoion*.

Previous studies have noted differences in the surface ultrastructure of two heteronchoinean monogeneans (*R. emarginata* and *P. gurnardi*) [8]. Uniquely among monogeneans, for example, *R. emarginata* possess a series of shallow pore-like infoldings at their free edge, and lack microvilli. The surface of the diclidophorid *P. gurnardi*, on the other hand, is similar to that of other monogeneans. The surface structure of *P. homoion* differs from both of these species in that no shallow pore-like infoldings or microvilli were recorded anywhere on the surface (except around the mouth cavity). Finally, while the apical membranes of *Atriaster* sp. [45] have some undulations and microvilli, similar to *E. soleae* or *A. elegans* [6], they are lacking in both *R. emarginata* [8] and *P. homoion*.

While the *P. homoion* neodermis possesses the same morphological properties in different body regions, slight differences could be discerned in the width of the outer syncytial layer and the amount of individual organelles. The outer syncytial layer on the middle forebody and cross-body region of *P. homoion*, for example, contains a higher proportion of V1 vesicles, in contrast to other body parts where V2 and V3 vesicles tend to predominate. Although the meaning of the vesicles has not yet been clarified in *Diplozoon* spp., these may have a storage function. The neodermal organelles and the role of the neodermis (tegument) in nutrient uptake by parasitic platyhelminths have previously been discussed by Dalton et al. [50]. In *P. homoion*, the outer syncytial layer around the apical mouth cavity, with its microvilli, ranging between 3 and 7 μm in total length. The different measurements recorded could be related to the parasite’s feeding technique, the mouth cavity having to be very flexible while sucking fish blood. Although the microvilli on the surface neodermis of the mouth have been discussed previously [14], their true function is still far from being understood, though it is likely to be related either to disruption of host tissue or uptake of host blood.

The clamps of *P. homoion* are covered with a very thin outer syncytial layer, which correlates well with results obtained for other monogenean species, such as *G. bivaginalis* and *C. leptogaster* [26, 27]. This reduction in external syncytial layer thickness probably reflects an adaptation related to the parasite’s life strategy, in that the clamps must be able to grab hold of tissue between the host’s gill lamellae in a very limited space.

### Clamps

While morphology and sclerite composition has frequently been studied in relation to species identification, morphological abnormalities, and taxonomic characterization of species of the family Diplozoidae [22, 51–53], there have been no ultrastructural studies on the clamps. Our findings on *P. homoion* correlate with observations on the clamps of other heteronchoinean monogeneans in that the clamp frame is usually formed of several electron-dense sclerites bound by radial muscle fibers, the surface is covered with a very thin outer syncytial layer, and nerve fibers are located close to the terminal and basal parts of the sclerites [26–28]. Association of nerve plexuses with the clamps has also been observed in *E. nipponicum* (a species closely related to *P. homoion*) using immunostaining and microscopic observation [54].

Early studies suggested that *Paradiplozoon* sp. sclerites were composed of chitin [55]; however, later work showed that the sclerites of *D. paradoxum* from the same family consisted of a resilin-like protein [32]. More recently, studies on the chemical composition of monogenean clamps have shown that they are formed of scleroproteins, probably stabilized by tyrosyl residues in dityrosine [29, 56].

Studies on the development and ultrastructure of *Gastrocotyle trachuri* clamp walls have detected numerous differentiating cells associated with the region around the epidermal infoldings and developing clamp [24, 57]. These cells produce cytoplasmic extensions that form a syncytial region around the epidermal infolding near the region of clamp formation. The same cells also produce groups of electron-lucent membrane-bound vesicles that pass along the extensions towards the region of clamp formation. In a previous study describing the mechanism of sclerite development [57], the authors noted formation of short hollow tubules at the start of the process, followed by an increasing number of tubules and development of areas of dense material within the developing sclerites, after which the sclerites became uniformly electron-dense prior to forming the clamps. We recorded electron-dense material migrating through the parenchyma to the sclerite surface, an observation supported by previous observations on *G. trachuri* [57], in which electron-lucent membrane-bound vesicles were observed moving along the differentiating cell extensions towards the region of clamp formation.

Here, we document for the first time, glandular cells containing secretory bodies at the base of the posterior median plate sclerite. Numerous gland cells have been observed opening ventrally into the ducts around the haptor suckers in *Polystomoides* spp. [58]. Cyton-containing secretory bodies of different sizes and shapes have been described between numerous membranous folds near the marginal hooklets in *Macrogyrodactylus congolensis* [59], although it is not thought that the glandular secretions play any role in attachment to the host’s skin by the marginal hooklets. The function of the haptoral gland cells at the base of the median plate sclerite, observed for the first time in our study, is still not fully understood and merits further attention. However, as three types of secretory products related to attachment have been observed in the anterior adhesive areas of two capsalid *Benedenia* monogeneans (*B. rohdei* and *B. lutjani*) [60], they could be associated with the process of attachment to the fish gills.

## Conclusion

Our work represents a first approach for examination the surface and attachments clamp ultrastructure in a species of the family Diplozoidae using TEM. Ultrastructural details were characterized for a range of tegumental compartments, including scales located on the hindbody, the outer surface layer, and the cytoplasmic bridge connecting the inner nucleated cell bodies and the outer syncytial layer. Our findings for *P. homoion* contradict previous information that the surface ridges are purely of neodermal origin; rather, the ridges show well-developed musculature and a neodermal outer layer localized externally to the basal lamina that covers the ridge’s surface. This study provides a unique comparison of the neodermis from different body parts for species from the family Diplozoidae. We were able to produce unique photo documentation of electron-dense material (responsible for sclerite development) migrating through the parenchyma to the sclerite surface. Similarly, we also documented the occurrence of glandular cells at the base of attachment clamps for the first time in a diplozoid species. The mechanisms involved in sclerite and glandular cell development merit further attention.
